# Benthic remineralization under future Arctic conditions and evaluating the potential for changes in carbon sequestration in warming sediments

**DOI:** 10.1038/s41598-024-73633-z

**Published:** 2024-10-07

**Authors:** Arunima Sen, Eric Jordà Molina, Thaise Ricardo de Freitas, Silvia Hess, Henning Reiss, Bodil A. Bluhm, Paul E. Renaud

**Affiliations:** 1 Department of Arctic Biology, The University Centre in Svalbard (UNIS), Longyearbyen, Norway; 2 Faculty of Biosciences and Aquaculture, Nord University, Bodø, Norway; 3 Department of Geosciences, University of Oslo, Oslo, Norway; 4 Department of Arctic Marine Biology, UiT The Arctic University in Norway, Tromsø, Norway; 5 Akvaplan-niva, Tromsø, Norway

**Keywords:** Carbon cycling, Carbon sequestration, Biological pump, Climate change, Benthic-pelagic coupling, Sediment oxygen demand (SOD), Ecology, Climate-change ecology, Community ecology, Ecosystem ecology

## Abstract

Benthic (seafloor) remineralization of organic material determines the fate of carbon in the ocean and its sequestration. Bottom water temperature and labile carbon supply to the seafloor are expected to increase in a warming Arctic and correspondingly, benthic remineralization rates. We provide some of the first experimental data on the response of sediment oxygen demand (SOD), an established proxy for benthic remineralization, to increased temperature and/or food supply across a range of Arctic conditions and regimes. Each factor significantly increased SOD rates (with different degrees of variability); however the largest increases were seen with both factors combined (50% to ten-fold increases), consistently across the four seasons and the spatial gradient covering shelf to deep basin included in our study. This ability of the Arctic benthos to process increased pulses of carbon suggests that increased sedimented carbon under warming conditions is likely to be utilized and processed, not accumulated, impacting carbon storage and decreasing the Arctic’s role as a global carbon sink.

**Supplementary Information:**

The online version contains supplementary material available at 10.1038/s41598-024-73633-z.

## Introduction

The biological pump determines the trajectory and fate of carbon at the global scale, and benthic remineralization is key to the process. Specifically, remineralization refers to the regeneration of organic matter into inorganic nutrients and carbon dioxide, which can then be fixed by primary producers into organic matter. In the ocean, remineralization takes place throughout the entire water column, but seafloor remineralization ultimately determines not just the cycling of carbon within the ocean^1–5^, but also its long-term storage and sequestration^6–9^.

In marine systems, rates of biological activity are often determined by temperature, and benthic remineralization rates are no exception^5^. Therefore, changes in bottom-water temperature related to global warming should induce changes in rates of benthic remineralization. In the Arctic, warming is particularly pronounced^10,11^, which means that benthic remineralization rates have the potential to change considerably. However, bottom-water temperature increases are not expected to be consistent across different regions of the Arctic. Inflow shelves receiving warmer waters, such as the Barents and Chukchi Seas, are expected to experience the greatest degree of bottom water warming, up to 5 °C over the next century^12–14^, and overall, shelves and coastal areas will likely experience warming bottom water to a larger extent than deep basins (+ 4 °C versus + 1–2 °C, respectively)^13,15,16^. Incorporating this regional and bathymetric variability into experimental studies of seafloor processes is important if predictions generated from the results are to be relevant.

The availability of labile organic matter is one of the other most important environmental factors driving remineralization rates for the Arctic benthos^5^. In the Arctic, food supply to the benthos is highly seasonal and tightly linked to surface primary production^2,17,18^. During the polar night, primary production is limited (though not entirely absent)^19,20^, but the return of sunlight stimulates two distinct pulses of high primary production^17,21^. The first occurs close to the onset of the return of sunlight when sea ice is still abundant. Under these conditions, sea ice algae can thrive and thick strands extending up to meters in length have even been seen trailing under the ice^22–26^. Significant phytoplankton production can also take place under the sea ice^27^, although large phytoplankton blooms occur in open ocean areas as spring progresses and sea ice melts with increasing day length and temperature^28–30^.

The decline of sea ice (by areal extent, thickness and amount of thick, multiyear ice)^31–35^ has extended the growing season for phytoplankton and expanded the areal extent and period of open water for phytoplankton blooms to occur across the Arctic and especially on interior shelves, including an increase in the frequency of autumn phytoplankton blooms^36–40^. Accordingly, annual phytoplankton primary production has increased across a large part of the Arctic, particularly on continental shelves^37,38^ and this trend is expected to continue due to (regionally) enhanced mixing and nutrient delivery due to more open water and more frequent storms^36,41–44^. Since both under-ice and autumn phytoplankton blooms are likely to peak before the seasonal ascent and after the seasonal descent of large zooplankton grazers, and since autumn blooms may be dominated by large, fast-sinking phytoplankton species^45^, the benthos will likely receive greater inputs of phytoplankton-based organic material in much of the future Arctic Ocean^46^.

**Fig. 1:**
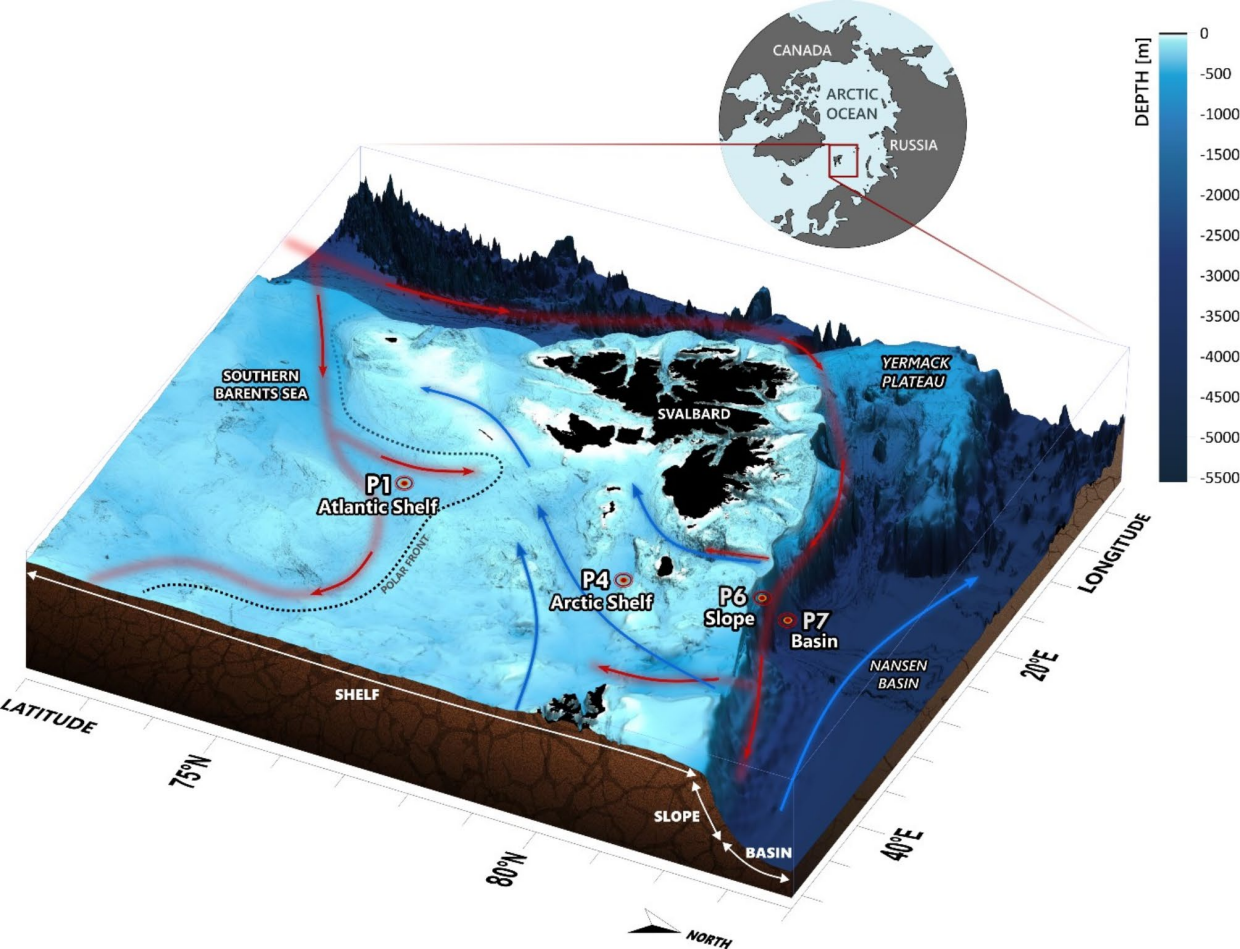
Map of the study area and the locations of the stations in this study. Station P1 (Atlantic shelf) is south of the polar front on the shelf, P4 (Arctic shelf) is north of the polar front on the shelf, P6 (Slope) is on the continental slope and P7 (Basin) is in the Nansen basin off the slope. Warm Atlantic water is shown with red arrows and cold, Arctic water is shown with blue arrows. Bathymetry is from General Bathymetric Chart of the Oceans (GEBCO) and currents are re-drawn from Vihtakari, 2020^93^. The map was created with Golden Surfer (v19, https://www.goldensoftware.com/) and Inkscape (v1.2.2, https://inkscape.org/).

**Fig. 2:**
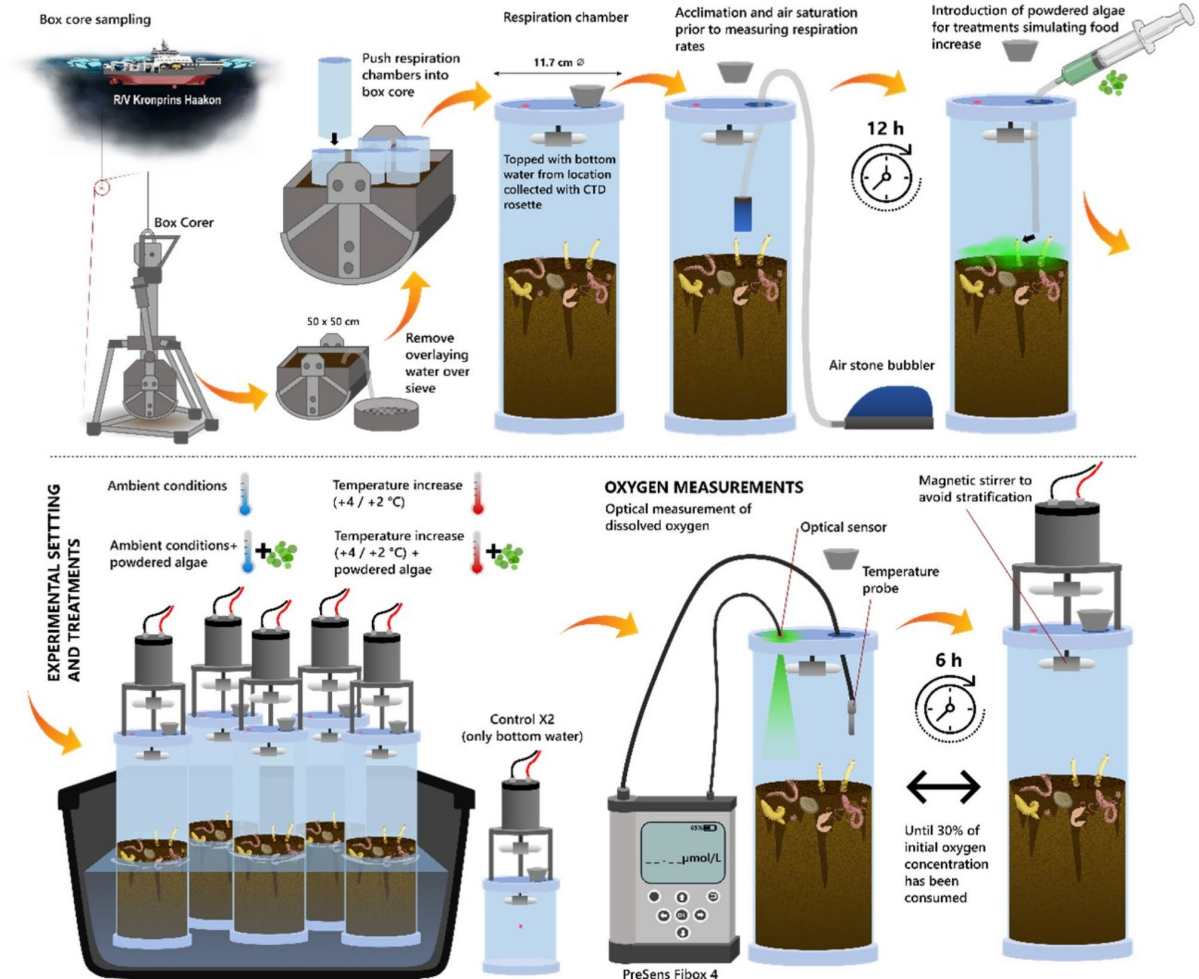
Illustration of the sampling process and the experimental setup. Treatments and controls were run in five replicates each (six during December).

The specific trajectories of primary production change will vary regionally^46^, but substantial summer sea ice loss has taken place in the central Arctic and the Barents Sea is expected to experience year round ice-free conditions by the end of the 21st century^47^. Therefore the above mentioned scenario of greater phytoplankton based carbon supply to the seafloor can be expected to play out in these regions, coupled with some of the largest increases in bottom water temperature^12,13^. The changes in both food supply and temperature at the seafloor may impact benthic remineralization rates in these regions and ultimately, the overall role of the Arctic as a global carbon sink. It is currently unclear whether increased temperature and food supply to the benthos in the central Arctic and Barents Sea will result in an enhanced biological carbon pump and a larger accumulation of carbon on the seafloor or whether benthic communities will be able to process the larger degree of incoming carbon. Quantifying and assessing changes in benthic remineralization rates in response to these predicted changes are hence necessary steps to understand the ecological impacts of climate change. Therefore, we quantified benthic remineralization rates across a transect from the shelf in the Barents Sea to the deep, abyssal plain of the Nansen Basin in four seasons (March, May, August and December) and in response to increased phytoplankton based food supply and warmer temperatures (Fig. 1). We further tested the combined effect of both increased temperature and food supply (see Table 1 and Fig. 2), since these two scenarios are expected to occur concomitantly. We hypothesized that higher temperature would result in higher remineralization rates at all stations and all seasons due to the established relationship between temperature and base metabolic rates. We also expected that the deeper stations (slope and basin) would exhibit stronger responses to increased food supply than shallower water shelf stations since food is comparatively more limited in deep water locations^5^. Finally, we expected that the response of remineralization rates to added phytoplankton based food supply would vary seasonally, depending on whether fresh phytodetritus had recently naturally arrived at the sea floor. We thus provide some of the most comprehensive data and insight on Arctic benthic remineralization within the context of climate change and projections for the future.

**Table 1:** Site and experiment details: treatments conducted (number of replicates in parentheses). All = all four treatments; of these, *amb* = ambient temperature, *algae* = ambient temperature with algae added, *warm* = increased temperature (+ 4 °C/+2°C). # cores = number of box cores, lat = latitude, lon = longitude, depth = water depth in metres, chl *a* = chlorophyll *a* (mg/m^2^), phaeo = phaeopigments (mg/m^2^), TOC = total organic carbon (%), N = nitrogen (%), Corg/N = TOC/N, avg. abundance = macrofaunal abundance (numbers of individuals), avg. biomass = wet weight biomass (grams), temp. = temperature of the *amb* treatment (asterisks indicate subzero ambient conditions). Values are averages from box cores for sediment (surface layer thickness in cm in parantheses) and from *amb* for fauna (full data sets in references). Values based on one replicate do not have standard deviations, however, standard deviations written as 0 refers to values < 0.001. Note that cores could not be taken successfully at the P1 Atlantic shelf location in December, the P7 Basin station in March and the P7 Basin station in December.

station/season/	treatments (reps.)	# cores	Lat. (°*N*)	Lon. (°E)	depth (m)	chl a (0–2 cm)	phaeo (0–2 cm)	chl a/ phaeo	TOC % (0–1 cm)	N % (0–1 cm)	Corg/*N*	avg. abundance	avg. biomass (g)	temp. (°C)
P1 Atlantic shelf- March	all (5)	3	76	31.2197	325	11.7	41.9	0.3	2.1 ± 0.1	0.3 ± 0.0	6.7	56.2 ± 13.1	1.52 ± 0.69	0.5
P1 Atlantic shelf- May	all (5)	3	76.0004	31.2215	326	12.0 ± 1.6	42.4 ± 10.9	0.3	2.0 ± 0.0	0.3 ± 0.0	6.9	58.2 ± 19.3	2.16 ± 0.87	1
P1 Atlantic shelf- August	all (5)	3	75.9997	31.2153	325	9.5 ± 3.9	29.6 ± 3.4	0.3	2.1 ± 0.1	0.3 ± 0.0	7.0	74.6 ± 25.5	2.24 ± 1.31	2
P4 Arctic shelf- March	all (5)	3	79.7712	33.6121	326	3.7 ± 1.1	31.3 ± 6.6	0.1	1.4 ± 0.0	0.2 ± 0.0	5.7	49.4 ± 7.7	1.82 ± 1.61	0.5
P4 Arctic shelf- May	all (5)	3	79.7508	34.0087	331	4.6 ± 0.2	27.6 ± 3.9	0.2	1.4 ± 0.0	0.2 ± 0.0	5.6	54.4 ± 12.0	2.02 ± 0.82	1
P4 Arctic shelf- August	all (5)	3	79.7457	34.0169	331	3.6 ± 0.7	19.5 ± 2.2	0.2	1.5 ± 0.1	0.3 ± 0.0	6.0	41.40 ± 6.0	2.67 ± 1.81	0*
P4 Arctic shelf- December	*amb* (6), *warm* (6)	3	79.759	33.995	335	4.1 ± 1.3	18.8 ± 3.4	0.2	1.5 ± 0.0	0.3 ± 0.0	5.7	41.2 ± 4.5	2.58 ± 1.31	0*
P6 Slope- March	all (5)	3	81.5467	30.8518	870	3.3 ± 1.4	21.4 ± 6.1	0.2	1.3 ± 0.0	0.2 ± 0.0	7.6	27.2 ± 8.4	0.13 ± 0.12	0
P6 Slope- May	all (5)	3	81.5369	30.8676	898	3.1 ± 0.5	25.6 ± 3.3	0.1	1.3 ± 0.0	0.2 ± 0.0	6.6	30.2 ± 8.1	0.05 ± 0.02	0.5
P6 Slope- August	*amb* (5), *algae* (5)	2	81.5452	30.8475	843	3.3 ± 0.9	18.8 ± 4.3	0.2	1.4 ± 0.0	0.2 ± 0.0	6.9	37.0 ± 9.7	0.25 ± 0.11	0
P6 Slope- December	*amb* (6), *warm* (6)	3	81.55	30.892	866	3.3 ± 1.2	15.8 ± 3.9	0.2	1.3 ± 0.1	0.2 ± 0.0	6.5	43 ± 4.5	0.01 ± 0.00	0.5
P7 Basin- May	all (5)	3	81.842	30.7571	3084	2.0 ± 0.5	15.3 ± 2.2	0.1	1.3 ± 0.0	0.2 ± 0.0	6.4	4.0 ± 1.9	0.06 ± 0.12	0.5*
P7 Basin- August	*amb* (5), *algae* (5)	2	81.7276	28.6712	2499	2.1 ± 0.5	14.7 ± 0.3	0.1	1.5 ± 0.0	0.2 ± 0.0	6.6	6.4 ± 2.4	0.06 ± 0.06	0.5*

## Results

**Fig. 3:**
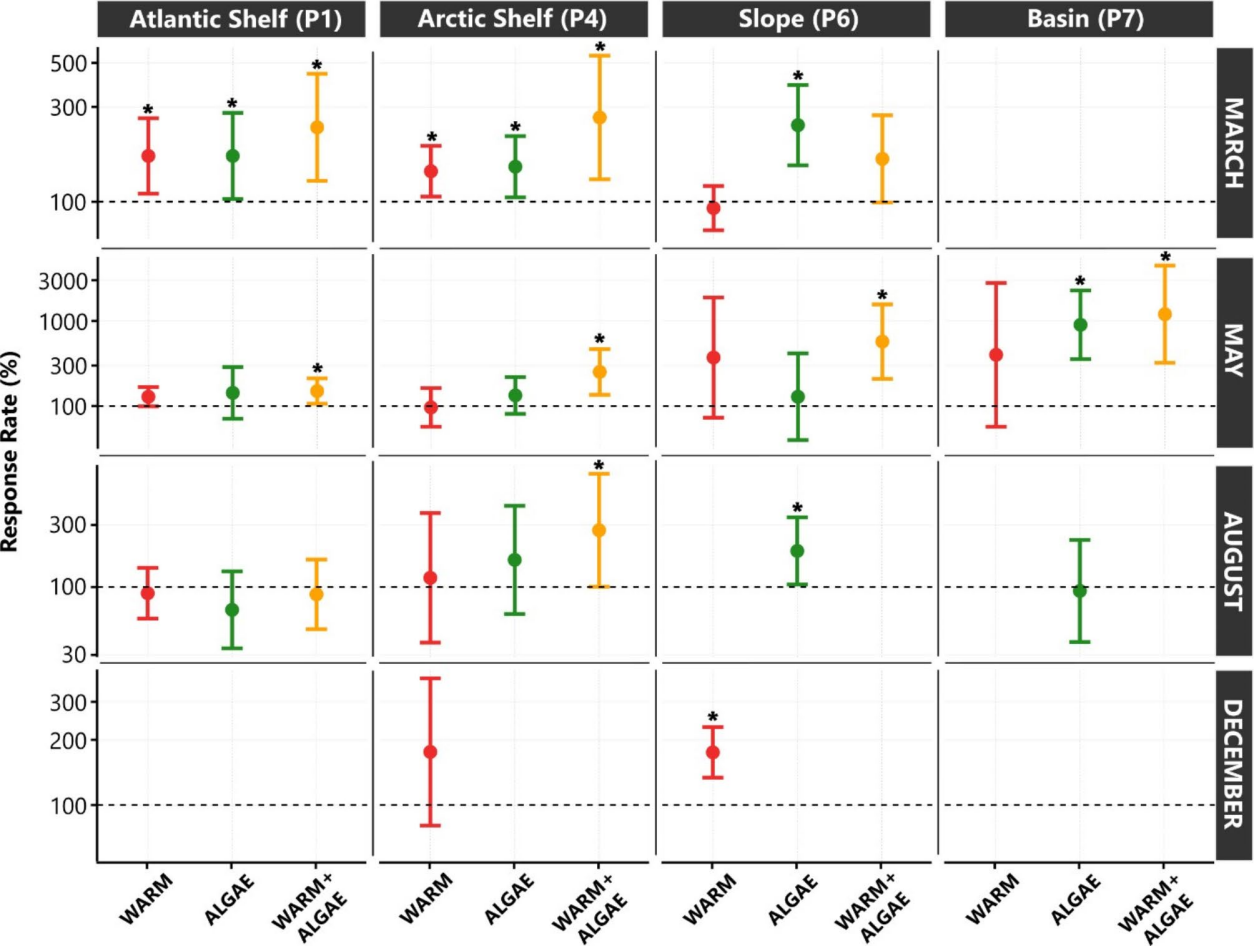
Sediment oxygen demand (SOD) rates shown as a response rate (%) of each treatment (x-axis) in relation to baseline SOD rates (*amb* treatment). Error bars represent 95% confidence intervals of the three manipulative treatments, and when they intersect the line at 100% there is no significant difference from baseline (*amb*) rates. Rates that were statistically significantly different from *amb* rates (i.e. above the 100% dashed line) are indicated with an asterisk. Month of sampling is indicated on the right of each row of figures. See Supporting Information Table 1 for rates for individual replicates.

In most cases, experimental treatments (*warm*,* algae and warm + algae*, see Fig. 2) exhibited higher SOD rates than baseline rates (Table 2). Within the *warm* treatment, higher increases were seen at the deep stations (up to 300% higher than baseline rates) than at the shelf stations (up to 77% higher than baseline rates, Table 2). The greatest increases however (approximately 300% higher than baseline at the Slope and Basin stations in May), were not always statistically significant. In fact, rates significantly higher than baseline were only recorded in March at the two shelf stations (70% and 43% higher than baseline rates at the Atlantic shelf and Arctic shelf respectively) and in December at the Slope station (74% higher than baseline rate) (Fig. 3).

**Table 2:** Sediment oxygen demand (SOD) rates for all stations in all seasons and across the four different treatments (mmol/m2/day). Numbers shown are average rates based on 5 replicates (6 in the case of December) ± standard deviation. For the three experimental treatments, i.e., with increased temperature and/or algae, percent changes compared to *amb* (ambient conditions) are written in parentheses with positive values indicating increased rates. Rates that were statistically significant from *amb* rates are indicated with an asterisk.

station/season	*amb*	*algae*	*warm*	*warm + algae*
P1 Atlantic shelf - March	3.0 ± 0.8	5.1 ± 0.9 (69%)*	5.1 ± 0.6 (70%)*	7.1 ± 1.6 (138%)*
P1 Atlantic shelf - May	2.8 ± 0.5	4.0 ± 1.6 (42%)	3.6 ± 0.2 (27%)	4.2 ± 0.6 (50%)*
P1 Atlantic shelf - August	5.7 ± 1.0	3.8 ± 1.4 (-33%)	5.1 ± 0.8 (-10%)	5.0 ± 1.4 (-12%)
P4 Arctic shelf - March	2.6 ± 0.4	3.9 ± 0.7 (52%)*	3.7 ± 0.5 (43%)*	8.6 ± 2.2 (230%)*
P4 Arctic shelf - May	2.7 ± 0.5	3.6 ± 1.0 (32%)	2.6 ± 0.9 (-6%)	6.8 ± 1.8 (146%)*
P4 Arctic shelf - August	3.4 ± 1.9	5.5 ± 1.3 (63%)	4.0 ± 1.9 (18%)	9.3 ± 2.0 (176%)*
P4 Arctic shelf - December	2.9 ± 1.3	n/a	5.1 ± 1.3 (77%)	n/a
P6 Slope - March	1.4 ± 0.2	3.4 ± 1.0 (154%)*	1.3 ± 0.3 (-1%)	2.3 ± 0.9 (68%)
P6 Slope - May	0.7 ± 0.7	0.9 ± 1.1 (38%)	2.6 ± 2.9 (300%)	4.0 ± 1.8 (508%)*
P6 Slope - August	1.9 ± 0.6	3.6 ± 1.1 (88%)*	n/a	n/a
P6 Slope - December	1.6 ± 0.2	n/a	2.8 ± 0.5 (74%)*	n/a
P7 Basin - May	0.3 ± 0.5	2.7 ± 1.1 (758%)*	1.2 ± 2.4 (275%)	3.6 ± 2.5 (1032%)*
P7 Basin - August	4.4 ± 1.8	4.1 ± 1.4 (-8%)	n/a	n/a

The *algae* treatment resulted in significantly different SOD rates from baseline rates in more instances: at least once for each station, and twice at the Slope station and these increases ranged from 54% higher than baseline rates (Arctic shelf in March) to 758% higher (Basin in May) (Fig. 3). During March, the *algae* treatment resulted in significantly higher SOD rates than baseline at all stations where experiments were conducted (Fig. 3). Similar to the *warm* treatment, the highest increases in SOD within the *algae* treatment were seen at the two deep water stations (up to 154 and 758% increases at Slope and Basin, respectively) (Table 2). At the Arctic shelf and Basin stations, the highest increases (88% and 758% higher than baseline respectively) occurred when the lowest chlorophyll *a* values were recorded in the sediment (August and May, respectively), but this was not the case for the other two stations (Tables 1 and 2).

Across all stations and seasons, the impact of the combination of increased temperature and food was much more pronounced than either factor taken individually (Table 2; Fig. 3). Other than at the Atlantic shelf station in August, the *warm + algae* treatment exhibited much higher rates than baseline rates (from 50% higher than baseline rates at the Atlantic shelf in May to over 1000% higher at Basin in May). Furthermore, in most cases, the *warm + algae* treatment rates were considerably higher than the *warm* and *algae* treatments individually (Table 2). In all but two cases, increases were statistically significant, i.e., the *warm + algae* treatment nearly always resulted in significantly higher than baseline SOD rates of higher magnitude than the other two treatments regardless of season (from 50% to ten times higher than baseline rates).

**Fig. 4:**
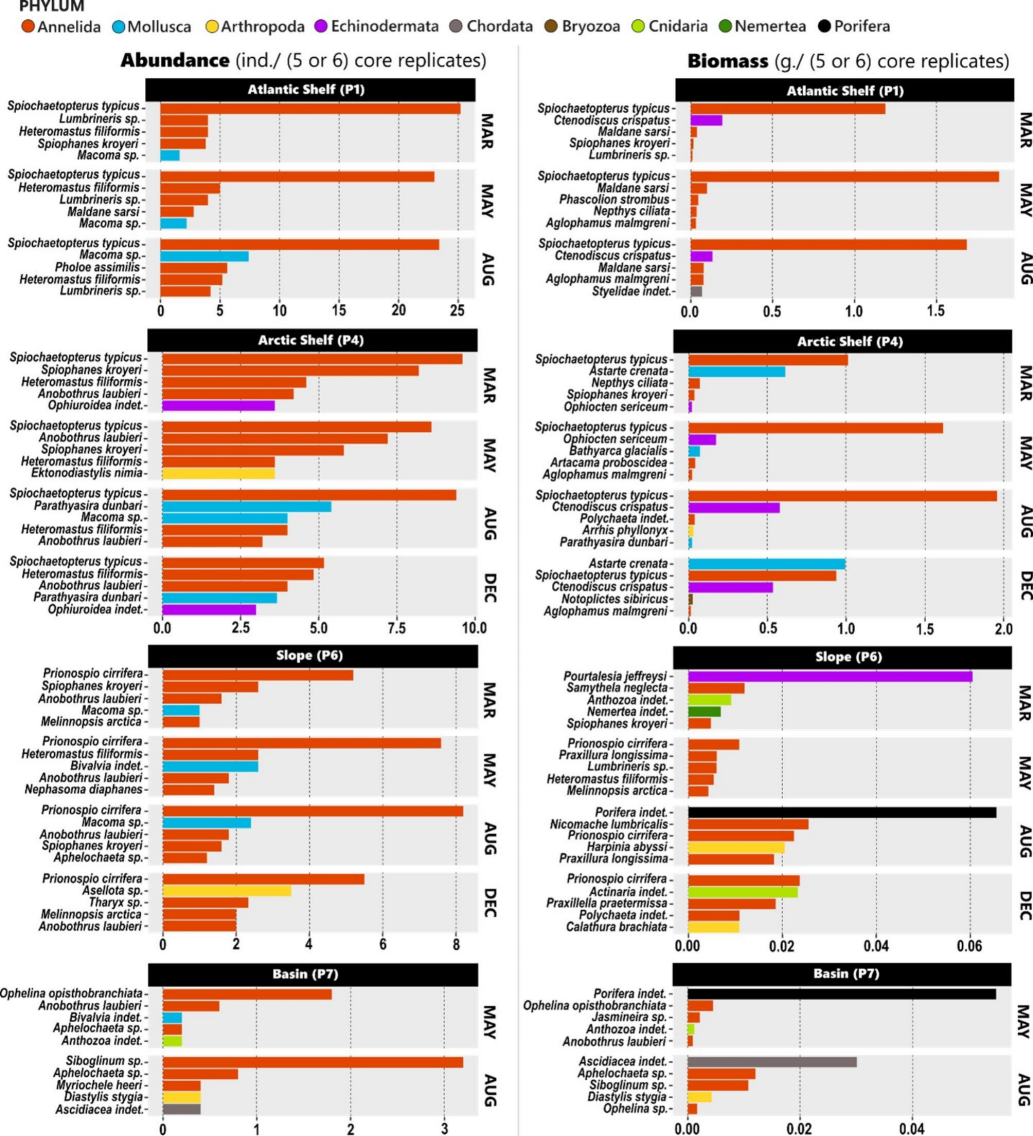
Abundance and biomass of the five taxa with highest abundance and biomass at each station during every sampling event (see Jordà-Molina et al., 2024^49^). Numbers for each taxon represent averages based on the ambient (*amb*) treatment replicate cores (December P6 biomass value is based on the *warm* treatment due to a problem with handling the samples).

Polychaetes were the most abundant group at all stations during each sampling event. Polychaetes largely dominated the biomass at the two shelf stations, however at the Slope and Basin stations, echinoderms, sponges and ascidians contributed the most to biomass (Fig. 4). Overall, macrofaunal abundance and biomass varied considerably between the shelf stations and the deeper water stations (Fig. 4; Table 1^48^,^49^). Despite this, neither environmental nor community parameters explained much of the spatial or seasonal variation in baseline SOD: none of the tested parameters exhibited a statistically significant impact on SOD (Table 3).

**Table 3:** Results of a multiple regression model testing chlorophyll *a* (chl *a*) in the top 2 cm, phaeopigments in the top 2 cm, total organic carbon (TOC) in the top 1 cm (%), total nitrogen (TN) in the top 1 cm (%), carbon to nitrogen ratio (Corg/N) in the top 1 cm, total abundance (numbers of individuals) of macrofaunal taxa and total biomass of macrofauna as predictors of sediment oxygen demand (SOD). Environmental parameters were averaged across the three replicates taken at each station, abundance and biomass were averaged from the incubation cores (*amb* treatment, and the *warm* treatment for P6 December biomass), and SOD was averaged from the incubation cores (*amb* treatment).

	coefficient	std. error	t-value	*p*-value
intercept	-9.36	70.70	-0.13	0.90
chl *a* (0–2 cm)	-0.56	0.49	-1.15	0.32
phaeopigments (0–2 cm)	-0.03	0.09	-0.34	0.75
TOC (0–1 cm)	4.27	47.32	0.09	0.93
TN (0–1 cm)	34.70	314.04	0.11	0.92
Corg/N (0–1 cm)	-0.07	10.60	-0.01	1.00
abundance	0.01	0.03	0.44	0.68
biomass	0.08	0.78	0.10	0.92
temperature	0.67	1.14	0.59	0.59

## Discussion

Temperature and labile carbon supply are the dominant drivers of benthic oxygen fluxes and organic matter remineralization in the Arctic Ocean^5,50^, and both of these are expected to increase as the Arctic warms^15,16,38,46^. Our experimental study, unprecedented in its temporal and spatial coverage, revealed modest impacts of increased temperature and food supply when tested individually, but more dramatic increases in benthic remineralization rates when the factors were combined. We discuss how these results have important implications for assessing the impact of a warming climate on Arctic carbon cycling and sequestration.

Temperature is well known as a primary driver of SOD in marine sediment^51–53^, including in cold regions such as the Arctic. Indeed, SOD has been shown to increase linearly with increasing temperature up to 19 °C in Svalbard fjords^53^. Thus, it was not surprising that our *warm* treatment led to increased SOD, although the response was more modest and variable than expected. Significantly higher rates over baseline were recorded only three out of the 11 times we conducted the *warm* treatment (Fig. 3). Unexpectedly, significant increases usually did not correspond with times when bottom water temperature was at its coldest at the different stations.

Similarly, the considerable spatial variability we recorded in the extent of SOD increase was somewhat unexpected. The highest increases were at the deeper stations (Slope and Basin) where SOD increased by up to three-fold (Table 2). At the two shelf stations, SOD was also enhanced over baseline rates, but less (up to 77%), despite a higher temperature increase. Ambient bottom-water temperature did not vary between all the stations by more than 2 °C, and varied even less between the Arctic shelf station and the deeper (Slope and Basin) stations (Table 1). Therefore, different ambient temperature alone is not a sufficient explanation for the deeper Slope and Basin stations exhibiting a greater response to increased temperature than the shelf stations. The relative contribution of different benthic community compartments to SOD could offer an explanation. Macrofauna is the primary contributor to SOD in Arctic shelf regions^1,2,54^ while in deeper locations, meiofauna and microbes contribute proportionally more to SOD rates^55^. We observed lower macrofaunal abundance and biomass at the Slope and Basin locations (Table 1^48,49^), and microbial activity is highly responsive to temperature^56,57^, which may partly explain why the Slope and Basin stations responded more strongly to increased temperature. However, some studies have also shown that even with increased microbial activity, SOD rates can remain relatively stable^50^. Be that as it may, we were not able to discern how much of the enhanced SOD in the *warm* treatment can be attributed to microbes or meiofauna versus macrofauna.

Taken together, these findings from our study and the literature suggest that other factors than temperature itself or meiofaunal/microbial activity must modulate the temperature response of sediment communities. We did not record a significant relationship between either TOC or chlorophyll *a* (measures of food quantity and quality) and SOD rates (Table 3), however, both TOC and chlorophyll *a* were consistently higher at the Atlantic shelf station than the other stations, as were baseline SOD rates. It was also at this station where the *warm* treatment showed a strong trend (though not statistically significant) more frequently than the other stations (Fig. 3). Therefore, we suggest that benthic temperature response is linked to food availability, specifically, that higher temperature does not enhance SOD unless sufficient quantities of labile organic material are present.

Benthic systems are known to be carbon-limited, and food availability is one of the most important factors determining SOD rates^5^. In the Arctic, seasonal variation in SOD has been observed to be directly related to organic material export to the seafloor^2–4^. Accordingly, we expected increases in SOD rates at times of low food supply to the benthos and correspondingly, times with low quantities of fresh phytodetrital material (indicated by chlorophyll *a* or the ratio of chlorophyll *a* to phaeopigments) in the sediment. We recorded significant increases to SOD rates in the *algae* treatment in March at all stations (except at the Basin station where this treatment was not conducted in March). Contrary to our expectations, levels of sedimentary pigments were close to annual maxima at each station and chlorophyll *a*/phaeopigment ratios did not indicate lower quality (low values) at the stations during this time (Table 1). Nonetheless this is actually a time when the quantity of labile carbon in the sediment was expected to be low since it is prior to spring blooms of ice algae or phytoplankton and well after any autumn phytoplankton blooms that might have occurred. Correspondingly, vertical flux of organic material was low during this period^58^.

There were two other instances when the SOD rates of the *algae* treatments were significantly higher than baseline: August at the Slope station, and May at the Basin station (Fig. 3). At all sampling points, the Slope and the Basin stations had lower quantities and proxies for quality of food than the two shelf stations (Table 1), and the consistency of our measurements (which are admittedly at low temporal resolution), do not indicate evidence of a strong flux of phytopigments to the seafloor at any time during the year. The depths of these Slope and Basin stations (900–3000 m), and the possibility of advective and consumption processes, can strongly dampen seasonal primary productivity signals^59^. Subsequently, these stations are likely food limited throughout the year and likely experience only episodic food pulses. Based on this, we should expect these stations to respond year-round to increased food supply. Thus the higher frequency with which algae addition increased SOD rates at the Slope and Basin stations could be due to those communities rapidly capitalizing on increased supply since they are otherwise perennially food deprived. In line with our expectations, the Slope and Basin stations exhibited additional significant responses to the *algae* treatments compared to the shelf stations.

The two periods when significant responses to added algae were not recorded at these two stations (Slope in May and Basin in August) may be linked to when chemosynthesis constituted a major nutrition source. As mentioned above, the specific locations of these Slope and Basin stations changed between sampling points due to issues with accessibility. Siboglinid worms, which use carbon fixed by chemolithoautotrophic symbiotic bacteria^60–62^ were present in high abundance, and even the dominant taxon, at the Basin station at these times (Fig. 4^48,48^). Neither siboglinid worms nor free-living chemosynthetic microbes feed directly on organic matter, therefore communities dominated by such groups might not immediately respond to increases in phytodetrital material. This needs to be kept in mind as glaciers retreat and expose methane-rich forefronts in fjords^63^ where chemosynthesis-based communities are likely to develop. Overall, the results of the *algae* treatment corresponded well with our expectations of responses being linked to periods/locations of low food availability and furthermore demonstrate the efficiency of the Arctic benthos in response to food inputs, as has been seen before^23,64–66^ .

Tests of the more realistic projection of a combination of higher temperature and greater food supply^37,67^ in Arctic sediment resulted in large and statistically significant increases in benthic SOD over baseline rates in nearly every experiment (Fig. 3). Specifically, within the *warm + algae* treatment, increases in SOD ranged from 50% over baseline rates (Atlantic shelf) to up to ten times baseline rates (Basin) (Table 2). The highest increases were seen at the two deeper water stations (Slope and Basin), but the Arctic shelf station also consistently displayed over 100% increases in SOD rates. SOD measured during individual studies of Arctic benthos varied by a factor of 2–10 both seasonally^2^ and spatially^5^, so this strong effect in our study is not unusually high. The response to the *warm + algae* treatment resulted in SOD rates that would consume the additional 912 mg m^−2^ of organic carbon added in 10–30 days across all stations and seasons studied. The amount of added algae, approximately equivalent to vertical carbon flux during 2–16 days of a spring phytoplankton bloom in the region, appears to be easily processed by seafloor communities across all depths and community types. It should be noted that we only mimicked phytoplankton derived organic material, large diatoms such as from sea ice algae can also be quickly consumed by the benthos, particularly deposit feeders^23,64^. Furthermore, processing time will vary depending on the degree of lability of the material added. Pure freeze-dried phytoplankton culture is expected to be quite labile, therefore this estimate represents a minimum time, however this regardless suggests the amount of presumably quite labile material added can be processed in an ecologically reasonable time period. Thus, it is clear that organic inputs from under-ice or autumn phytoplankton blooms are likely to be processed and *not* likely to accumulate and get buried in the sediment in a future Arctic.

Pelagic-benthic coupling is known to be particularly tight on Arctic shelves^68,69^, and our results demonstrate that benthic communities in the Arctic deep-sea also respond rapidly to increased food and temperature. Changes in primary productivity regimes, links with grazer phenology, processes governing vertical flux, and the ability for the benthic community to respond all influence the efficiency of the biological pump^23,66,70,71^ and are likely to be affected by ongoing climate change^68,72,73^ which will influence the balance between organic matter recycling and carbon burial. Although estimates of the proportional roles of continental shelves and margins versus the deep sea for carbon burial vary by study^6,7,9^, it is established that the global seafloor represents a major long term sink for carbon. In the deep sea, only 1–2% of the organic material reaching the seafloor gets remineralized due to remineralization rates positively correlating with carbon fluxes which are low in the deep sea^74^.Carbon turnover rates tend to be higher on shelves^50,55^ however some shelf habitats such as fjords also exhibit globally high rates of carbon burial due to high carbon inputs from both terrestrial catchments and marine surface production^75,76^. Among fjords, carbon burial rates can differ based on the proportion of depositional organic material originating from terrestrial versus marine sources which in turn is linked to water masses and hydrographical regimes that are expected to change with the climate^76^. A number of factors therefore can determine the quantity and lability of organic material reaching different parts of the seafloor and subsequent burial/remineralization rates which are sensitive to climate related changes. Our results offer insight into how benthic processes may regulate carbon burial under climate-change scenarios.

Predicted large increases in flux of labile organic matter at the seafloor as tested in this study may even lead to a reduction in current sedimentary carbon inventories, although, as mentioned above, responses will differ by region, location and habitat type. Organic priming, a process by which the presence of labile material enhances processing of more recalcitrant organic material such as detritus or terrestrially derived material, is suggested to be more important than recognized, particularly in areas with high carbon input^77^. This has yet to be explored in the Arctic, but given the rapid response of the benthos to projected inputs of fresh organic material in this study, it is conceivable that organic priming could play an important role in future carbon dynamics. Lower carbon burial rates as well as the potential for release of already stored carbon can therefore reduce the ocean’s seafloor sequestration capacity, with significant consequences for climate-regulation services provided by the marine system.

Warming in the Arctic has progressed at a pace underestimated by most models^11^ and the effect of this on the Arctic’s role in carbon transfer and storage has been examined with respect to factors such as the decline of sea ice and albedo^78,79^, much more than in terms of alterations of benthic processes. By conducting experiments targeting the response of the benthos to both increased food and temperature across a comprehensive seasonal and spatial spectrum, we provide some of the first concrete insight into how the Arctic benthos could potentially compromise the extent to which the Arctic functions as a global carbon sink.

Methods.

Incubations and sediment sampling were conducted from the research vessel *Kronprins Haakon* as part of the Nansen Legacy project (arvenetternansen.com) during four cruises: August 2019, December 2019, March 2021 and May 2021. The March and May cruises were planned to directly follow the 2019 cruises but had to be delayed to 2021 due to the Covid-19 pandemic (see Table 1 for details on sampling).

## Study area and sediment sampling

Sampling was conducted at four stations (P1, P4, P6 and P7) that represent different environmental and bathymetric regimes (Fig. 1). P1 is located on the shelf at a water depth of about 325 m (Fig. 1), and is within warm, salty Atlantic water (bottom water temperature was measured to be 0.5–2 °C during our sampling). Surface waters are perennially ice free in this location and we therefore refer to it as the Atlantic shelf station. P4 is further north (330 m water depth) and is characterized by colder, Arctic water (bottom water temperature between − 1 and + 1 °C when we sampled) and was covered by sea ice during each season sampled, and we refer to it as the Arctic shelf station. P6 is located on the continental slope (Slope station) at a depth of around 850–900 m, where we observed thick (usually at least 1 m) sea ice during all our sampling efforts. The steepness of the slope resulted in sampling locations varying somewhat in position and depth (by 50 m) across the four sampling periods. P7 is in the Nansen Basin, at approximately 3000 m water depth. Year-round ice cover (excluding open water leads) made it logistically difficult to return to the same coordinates for P7 on every cruise; however, we nonetheless targeted deep-water locations at the edge of the Nansen Basin and refer to this station as the Basin station.

At each station, sampling was conducted with a box core (50 cm x 50 cm x 60 cm). Usually, three replicate box cores were taken at each station, although in some cases, time and other constraints restricted us to two replicates (Table 1). Once box cores were on deck, overlying water was gently siphoned off to sample the sediment surface. Only box core samples with undisturbed sediment were used. From each box core, subcores (11.7 cm inner diameter and approximately 20 cm deep) were taken for incubations (Fig. 2). Additionally, from each box core, subcores for chlorophyll *a*, phaeopigments, total organic carbon (TOC) and total nitrogen (TN) were taken and sliced at 1 cm intervals. All slices were frozen on board at -20 °C until they were processed in the lab^80–87^.

## Oxygen uptake incubations

Bottom water was collected at each station using the CTD/rosette and was carefully added to the incubation subcores without disturbing the sediment surface. Sediment heights in each subcore were measured to calculate the volume of overlying water. Air stones were inserted and incubation subcores were kept in the dark in temperature-controlled rooms for 12 h to allow for the benthos to acclimate and overlying waters to reach saturation of dissolved oxygen (Fig. 2). We recognize that pressure effects from running on-board incubations on sediments from the deep sea (> 1000 m) can bias rate measurements (reviewed by Smith and Hinga, 1983^88^, but see Pfannkuche and Thiel, 1987^89^). Since statistical comparisons for assessing the impact of food and temperature on respiration rates were limited to among-treatment effects within each station, we feel the pressure effect does not substantially impact our conclusions, although the specific rates measured may have been affected.

After the acclimation period, air stones were removed and the incubation cores were topped off with bottom water and sealed. Lids with slowly rotating magnets prevented stratification of the water within the cores. Core lids were also fitted with calibrated (0% and 100% saturation) PreSens oxygen measuring spots, and oxygen measurements were taken every six hours from each core using the Fibox 4 optical sensor (PreSens Precision Sensing GmbH) (Fig. 2). Incubations lasted until approximately 30% of the oxygen was consumed.

Four experimental treatments were imposed. Treatment ambient (*amb*) was carried out at near-in situ bottom water temperature. Since we were limited to a minimum operational temperature in the cold room of 0 °C, the ambient treatment was slightly higher than in situ temperatures when bottom water conditions were below zero (this occurred four times across the entire study, see Table 1). Treatment *algae* was kept at ambient temperature, and 30 g of dried, ground cultured microalgae (*Dunaliella tertiolecta* in 2019 and *Thalassiosira weissflogii* in 2021, naturally occurring algae in the Barents Sea) was added to the cores to mimic enhanced phytoplankton export to the benthos. Algae added is the equivalent of 912 mg C m^−2^, or approximately 2–16 days of sinking carbon flux in spring/summer in the western Barents Sea^3,58,73^ .

Treatment *warm* was carried out at warmer than ambient temperatures, either 4 °C higher than ambient for the two shelf stations, or 2 °C higher than ambient for the deeper Slope and Basin stations. These two temperature increases were chosen to represent projected increases in bottom water temperature by 2099 ^13,16^. Treatment *warm + algae* combined both warmer temperature and algal input (Fig. 2).

Each treatment included five or six replicate cores. Subcores from replicate box core deployments were interspersed among treatments so that each treatment consisted of replicate cores from the three different box core samples. The algal treatment experiments were not conducted during the December cruise since increased algal input is not expected during polar night periods. Due to problems with box core deployments and sea-ice conditions, not all treatments could be carried out at all stations in every season (Table 1).

In each temperature-controlled room, two chambers with only bottom water were incubated in the same manner as the sediment cores (during August only one core in each room). These served as controls to estimate the oxygen consumption rate of the bottom water pelagic community (see section on SOD measurements).

After incubations were completed, all core contents were sieved over a 0.5 mm mesh and the retained animals were fixed in 4% buffered formaldehyde with Rose Bengal and stored in the cold until sorting was done at Nord University, Akvaplan-niva and Institute of Oceanology, Polish Academy of Sciences (IOPAN) labs. From the *amb* treatment, total macrobenthic abundance and biomass were measured for each replicate core.

## Sediment oxygen demand (SOD) measurements and statistical analyses

For each core, oxygen concentrations were plotted against time and carefully examined for sudden peaks or drops that could have occurred due to mechanical problems (stopping of stirring) or oxygen levels dropping below 70% of original values. Data collected after such events and time points were discarded and not used in calculations. Oxygen concentrations (mmol) measured during the course of the incubations were fitted to a linear model and the (negative) slope of this model represented the rate of oxygen consumption for that whole core. In order to estimate oxygen uptake rates for the benthos only, the average oxygen consumption rates from the control chambers (bottom water only) were subtracted from each core’s measured oxygen uptake rate. Based on the surface area of the cores and volume of water in the core tube, SOD was calculated as mmol O_2_ m^−2^ d^−1^.

Our primary goal was to examine the effect of warming and/or algal addition treatments on SOD, and whether responses varied by station location and season. For this purpose, log-transformed response ratios (LnRR) were calculated based on Hedges et al. 1989 ^90^ and Vihtakari et al. 2016 ^91^. Log differences between *amb* (baseline rates) and the experimental treatments (*warm*,* algae and warm + algae*) were calculated. Response ratios (RR) were then back calculated from the log responses and expressed as percentages. Therefore 100% represents a value equal to the *amb* treatment, or baseline rates, and the effect of each treatment can be visualized whereby responses are deemed significantly different from *amb* if the 95% confidence intervals do not cross the 100% value. We further tested the effect of sediment parameters (chlorophyll *a*, phaeopigments, TOC, C/N) and macrofaunal abundance and biomass in driving those baseline rates through a multiple regression model. Statistical analyses were carried out in R with the tidyverse and AICCmodvag packages^92^.

## Electronic supplementary material

Below is the link to the electronic supplementary material.

Supplementary Material 1

## Data Availability

Data used in this study have been published and are publicly available at the following locations: https://gbif.imr.no/ipt/resource? r=aen_benthic_macrofauna#anchor-description (macrofauna data), https://doi.org/10.21335/NMDC-1821375519, https://doi.org/10.21335/NMDC-350572235, https://doi.org/10.21335/NMDC-490057692, https://doi.org/10.21335/NMDC-799257283 (sediment carbon and nitrogen data), https://doi.org/10.11582/2023.00030, https://doi.org/10.11582/2023.00031, https://doi.org/10.11582/2023.00032, https://doi.org/10.11582/2023.00033 (sediment chlorophyll a and phaeopigment data). Published datasets are cited throughout the article. The individual replicates of SOD rates are provided as supporting information. The R scripts used for retrieving the data from the public repositories, extracting the subsets we used for our analyses, and the scripts for conducting the statistical analyses are published and available here: https://zenodo.org/records/11202836.
